# Orthogonal targeting of EGFRvIII expressing glioblastomas through simultaneous EGFR and PLK1 inhibition

**DOI:** 10.18632/oncotarget.3996

**Published:** 2015-05-05

**Authors:** Ying Shen, Jie Li, Masayuki Nitta, Diahnn Futalan, Tyler Steed, Jeffrey M. Treiber, Zack Taich, Deanna Stevens, Jill Wykosky, Hong-Zhuan Chen, Bob S. Carter, Oren J. Becher, Richard Kennedy, Fumiko Esashi, Jann N. Sarkaria, Frank B. Furnari, Webster K. Cavenee, Arshad Desai, Clark C. Chen

**Affiliations:** ^1^ Center for Theoretical and Applied Neuro-Oncology, Moores Cancer Center, Division of Neurosurgery, University of California San Diego, La Jolla, CA, USA; ^2^ Department of Pharmacology and Chemical Biology, Shanghai Jiao Tong University School of Medicine, Shanghai, China; ^3^ Collaborative Innovation Center for Translational Medicine, Shanghai Jiao Tong University School of Medicine, Shanghai, China; ^4^ Department of Radiation Oncology, Dana-Farber Cancer Institute, Boston, MA, USA; ^5^ San Diego Branch, Ludwig Institute for Cancer Research, University of California San Diego, La Jolla, CA, USA; ^6^ Departments of Pediatrics and Pathology, Preston Robert Tisch Brain Tumor Center, Duke University Medical Center, Durham, NC, USA; ^7^ Centre for Cancer Research and Cell Biology, Queen's University of Belfast, Belfast, UK; ^8^ The Sir William Dunn School of Pathology, University of Oxford, Oxford, UK; ^9^ Department of Radiation Oncology, Mayo Clinic, Rochester, MN, USA

**Keywords:** EGFR, EGFRvIII, glioblastoma, synthetic lethality

## Abstract

We identified a synthetic lethality between PLK1 silencing and the expression of an oncogenic Epidermal Growth Factor Receptor, EGFRvIII. PLK1 promoted homologous recombination (HR), mitigating EGFRvIII induced oncogenic stress resulting from DNA damage accumulation. Accordingly, PLK1 inhibition enhanced the cytotoxic effects of the DNA damaging agent, temozolomide (TMZ). This effect was significantly more pronounced in an *Ink4a/Arf(−/−)* EGFRvIII glioblastoma model relative to an *Ink4a/Arf(−/−)* PDGF-β model. The tumoricidal and TMZ-sensitizing effects of BI2536 were uniformly observed across *Ink4a/Arf(−/−)* EGFRvIII glioblastoma clones that acquired independent resistance mechanisms to EGFR inhibitors, suggesting these resistant clones retain oncogenic stress that required PLK1 compensation. Although BI2536 significantly augmented the anti-neoplastic effect of EGFR inhibitors in the *Ink4a/Arf(−/−)* EGFRvIII model, durable response was not achieved until TMZ was added. Our results suggest that optimal therapeutic effect against glioblastomas requires a “multi-orthogonal” combination tailored to the molecular physiology associated with the target cancer genome.

## INTRODUCTION

It has long been observed that expression of potent oncogenes frequently induces cell cycle arrest, death, or senescence [1]. From these observations emerged the notion that oncogenic activation is accompanied by “oncogenic stress” that is prohibitive to cell survival or proliferation [2]. In this paradigm, cellular adaptation to these stresses through compensatory events is a requisite for neoplastic transformation and tumor viability [3, 4]. Implicit within this framework is the concept that oncogenic cell states are sensitive to inhibition of these compensatory mechanisms [5]. These forms of synthetic lethality have been born out in a number of studies demonstrating selective ablation of cells expressing potent oncogenes through inhibition of compensatory mechanisms [6, 7]. Notably, many oncogenic drivers induce DNA accumulation as a form of oncogenic stress [8, 9], requiring up-regulation of DNA repair or altered DNA damage response (DDR) for cell viability [10, 11].

Importantly, up-regulation of DDR has not been reported as resistance mechanisms for inhibitors targeting oncogenic proteins [12, 13]. Similarly, cancer cells that emerged after treatment with DNA damaging agents have not exhibited significant alteration in oncogenic signaling [14]. These observations suggest the possibility of imposing parallel and “orthogonal” [4] selections against the oncogenic state by simultaneous inhibition of DDR, thereby exacerbating the deleterious effects of oncogenic stress, and inhibition of the pertinent oncoprotein. Cancer cells subjected to such combination will need to independently evolve resistance mechanisms to restore oncogenic signaling as well as adaptive mechanisms to oncogenic stress. We tested this hypothesis in the context of an oncogenic form of Epidermal Growth Factor Receptor (EGFR), termed EGFRvIII [15].

EGFRvIII is a recurrent oncogenic variant found in 25-64% of glioblastomas [16-18], the most common form of primary brain cancer [19, 20]. This variant harbors a deletion that spans exons 2-7 of EGFR, a region that encodes a significant portion of the EGFR extracellular ligand-binding domain [17]. EGFRvIII is essential for glioblastoma initiation as well as proliferation [15]. In clinical specimens, EGFRvIII expression is typically detected in only a small portion of glioblastoma cells [3, 17]. However, clinical efficacy has been reported with selective ablation of this cell sub-population [21, 22], suggesting these EGFRvIII expressing glioblastoma cells exert potent effect on the biology of the overall tumor mass [23].

Our previous study demonstrated that EGFRvIII expression in glioblastomas induced oncogenic stress in the form of excessive DNA damage accumulation [11]. Here, we report that polo-like kinase 1 (PLK1) compensates for this stress through modulation of homologous recombination (HR). The tumoricidal and TMZ-sensitizing effects of the PLK1 inhibitor, BI2536, depends on the intrinsic physiology of the glioblastoma and correlated with the endogenous levels of DNA damage. Supporting our hypothesis, BI2536 augmented the ablative effects of Gefitinib, an EGFR inhibitor. However, durable response in a *Ink4a/Arf(−/−)* EGFRvIII model was not observed until temozolomide (TMZ), a DNA alkylating agent and the standard-of-care chemotherapy for glioblastoma, was added to the regimen. We termed this strategy “multi-orthogonal” because each component of the regimen acts in an orthogonal manner relative to others.

## RESULTS

### Synthetic lethality between EGFRvIII expression and PLK1 inhibition

**Figure 1:**
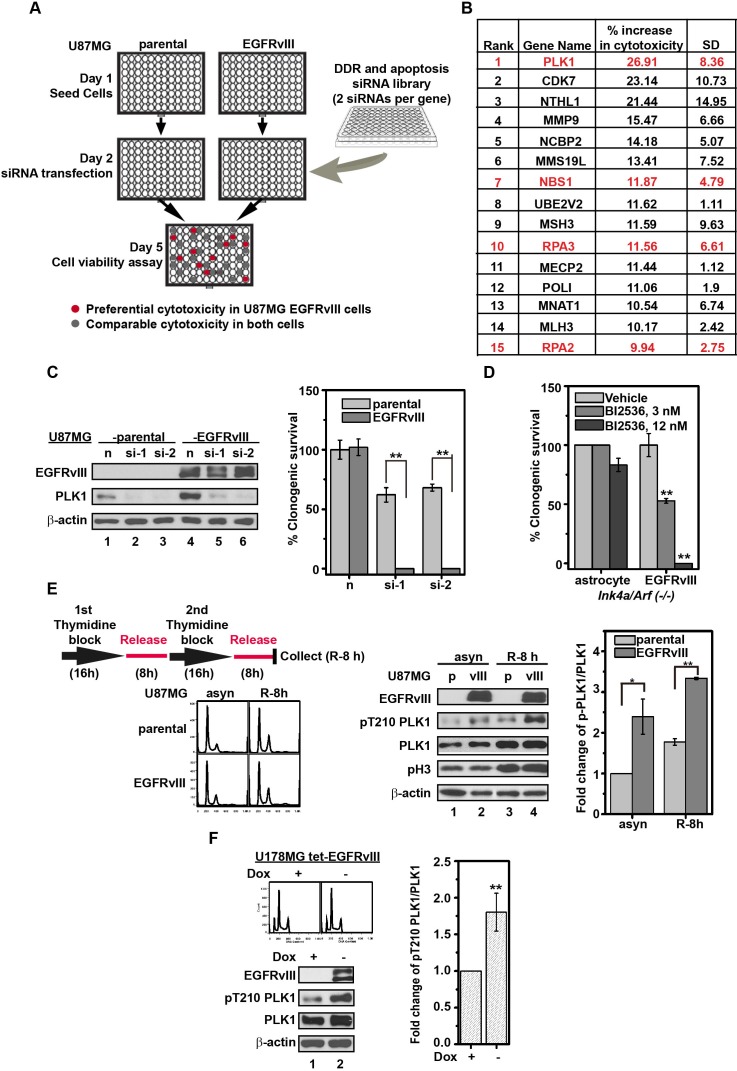
Silencing or inhibition of PLK1 is preferentially toxic to U87MG EGFRvIII cells **A.** Schematic depiction of siRNA library screen. **B.** The top siRNA targets that, when silenced, were preferentially toxic to the U87MG EGFRvIII cells relative to the U87MG parental cells. Red: DDR genes involved in HR. The “% increase in cytotoxicity” was calculated based on the mean of two independent experiments. **C.** (left) Immunoblot of PLK1 following knockdown with two independent siRNAs, siPLK1-1 (si-1) and siPLK1-2 (si-2). Negative control siRNA is indicated as n. Whole cell lysates were collected 48 h after siRNA transfection. (right) Clonogenic survival following PLK1 siRNA transfection in U87MG parental and U87MG EGFRvIII cells. **, *p* = 0.0092 and 0.0019 respectively. **D.** Effect of BI2536 on murine *Ink4a/Arf(−/−)* EGFRvIII cells and parental *Ink4a/Arf(−/−)* astrocytes. Clonogenic survival was determined after 14 days treatment. **, *p* = 0.0027 and 0.0036 respectively. **E.** (left upper) Schematic depiction of cell synchronization by double thymidine blocking (DTB). (left lower) Cell cycle distribution of U87MG parental and U87MG EGFRvIII cells after DTB. (middle) Representative immunoblots of whole cell lysates derived from synchronized and asynchronous U87MG parental (p) and U87MG EGFRvIII (vIII) cells. (right) Quantitative densitometric assessment of pT210 PLK1 was normalized to the total PLK1 after correcting for protein loading using β-actin level respectively. asyn, asynchronized cells; R-8 h, synchronized cells released from DTB for 8 h. *, *p* = 0.044; **, *p* = 0.0014. **F.** (left upper) Cell cycle distribution of U178MG tet-EGFRvIII cells with or without doxycycline (Dox) treatment at 1 μg/mL for 96 h; (left lower) Representative immunoblots of U178MG tet-EGFRvIII cell lysates with or without Dox treatment; (right) Quantitative densitometric assessment of pT210 PLK1 as above described. **, *p* = 0.0058. The densitometric results represent the average of three experiments, shown as mean±SD.

To identify DDR genes required for compensating EGFRvIII-associated oncogenic stress, we screened 714 siRNAs directed against 357 DDR genes for preferential toxicity to EGFRvIII over-expressing U87MG (U87MG EGFRvIII) cells relative to its parental cells (Figure 1A). Top candidates were highly enriched for DDR genes involved in homologous recombination (HR) (Figure 1B, shown in red). The top scoring hit, PLK1, was selected for subsequent validation because of the availability of clinical grade PLK1 inhibitors [11]. To exclude the possibility of off-target effects, two additional PLK1 siRNAs were tested, and both exerted preferential toxicity to the U87MG EGFRvIII cells (0% viable) relative to U87MG parental cells (50-60% viable, Figure 1C). Moreover, BI2536, a PLK1 inhibitor, completely ablated *Ink4a/Arf(−/−)* EGFRvIII cells while minimally affecting the parental *Ink4a/Arf(−/−)* astrocytes at a 12 nM concentration (Figure 1D).

### Hyper-activation of PLK1 in EGFRvIII expressing glioblastomas

The synthetic lethal interaction suggests that EGFRvIII expressing glioblastomas harbored heightened requirement of PLK1 activity. Consistent with this hypothesis, we found increased levels of an active form of PLK1 (pT210 PLK1) in U87MG EGFRvIII cells relative to U87MG cells. The increase in pT210 PLK1 was found in both synchronous and asynchronous cell populations (Figure 1E), indicating that the difference was independent of cell cycle progression. Similar results were observed in U178MG human glioblastoma cells conditionally expressing EGFRvIII (U178MG tet-EGFRvIII) (Figure 1F). These results suggest that EGFRvIII expressing human glioblastomas harbored higher levels of active PLK1.

### PLK1 inhibition enhanced accumulation of mitotic DNA damages

**Figure 2:**
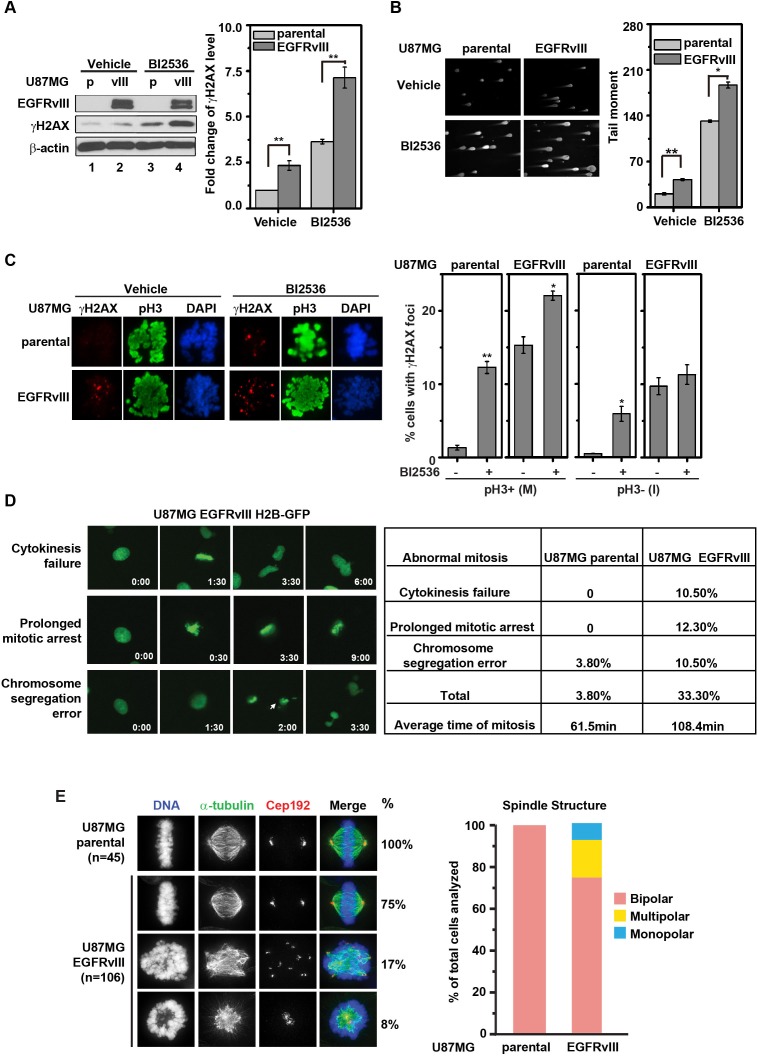
BI2536 treatment leads to increased DNA damage accumulation in U87MG EGFRvIII cells **A.** (left) Representative immunoblots of U87MG and U87MG EGFRvIII cells treated with vehicle or 25 nM BI2536 for 24 h. (right) Quantitative densitometric assessment of γH2AX level normalized to β-actin. **, *p* = 4.92×10^−5^ and 0.0012 respectively. **B.** (left) Representative comet staining images in U87MG EGFRvIII cells and U87MG parental cells after BI2536 treatment (25 nM for 24 h). (right) Quantitative assessment of comet tail moment. **, *p* = 0.01; *, *p* = 0.037. **C.** U87MG EGFRvIII cells and U87MG parental cells were stained for γH2AX and pH3 after 24 h treatment with 5 nM BI2536 or control. (left) Representative immunofluorescence staining. (right) Quantitation of γH2AX foci in pH3+ (M) and pH3- (I) cells. Approximately 100 cells were scored. **, *p* = 0.0063; *, *p* = 0.035 and 0.036 for vehicle control respect to BI2536 treated samples correspondingly. **D.** (left) Representative images of aberrant mitotic progression in U87MG EGFRvIII H2B-GFP cells. Time elapsed was indicated in the right lower corner (h:min). Arrow marks a lagging chromosome (see arrow at hour 2:00). (right) Frequencies of aberrant mitotic events in U87MG parental and U87MG EGFRvIII cells. Length of mitosis was scored from the onset of prophase to the onset of anaphase. Approximately 100 cells were scored, respectively. **E.** Aberrant multipolar and monopolar mitotic spindles in the U87MG EGFRvIII cells. (left) U87MG parental and U87MG EGFRvIII cells were stained for α-tubulin and the centrosome protein Cep192. (right) Quantification of bipolar, multipolar, and monopolar mitotic figures in U87MG parental and U87MG EGFRvIII cells.

A previous genome-wide siRNA screen revealed that PLK1 silencing led to a significant induction in γH2AX formation, suggesting PLK1 suppressed DNA damage accumulation [24]. In the context of our previous finding that EGFRvIII expression is associated with an elevated level of DNA damage [11], we hypothesized that PLK1 prevented the lethal accumulation of DNA damage in EGFRvIII expressing glioblastomas. Supporting this hypothesis, PLK1 inhibition by BI2536 induced a ~ 3-fold increase in γH2AX accumulation; this increase was further magnified by EGFRvIII expression (by an additional 2-3 fold, Figure 2A). Similar results were observed using the Comet assay (Figure 2B).

Given PLK1's role in mediating cellular adaptation [25], we explored the possibility that PLK1 inhibition may alter the cell-cycle distribution of DNA double-strand breaks (DSBs). U87MG parental or EGFRvIII cells were co-stained with the antibodies against γH2AX and histone H3 phosphorylated at serine 10 (pH3) in order to discriminate DSBs present during mitosis (pH3+) versus interphase (pH3-) respectively. EGFRvIII expressing cells exhibited increased γH2AX foci throughout the cell cycle (Figure 2C). BI2536 treatment of U87MG EGFRvIII cells increased the proportion of pH3+ cells with γH2AX foci, without significantly altering the proportion of pH3- cells with γH2AX foci (Figure 2C). Similar results were observed with flow cytometric analysis of cells co-stained with antibodies against γH2AX and pH3 (Supplemental Figure 1). These results suggest that PLK1 inhibition predominantly enhanced DSBs accumulation during the mitosis of the cell cycle.

### EGFRvIII expression is associated with aberrant mitotic progression

We next tested whether the accumulation of mitotic DNA damage in U87MG EGFRvIII cells was associated with aberrant mitotic progression. Time-lapse imaging of H2B-GFP labeled cells indicated that, on average, U87MG EGFRvIII cells required 108.4 minutes to complete mitosis, compared to the 61.5 minutes required for U87MG parental cells. Moreover, 33.3% of the U87MG EGFRvIII cells underwent aberrant mitosis (compared to 3.8% of U87MG parental cells, Figure 2D). Further analysis revealed that U87MG EGFRvIII cells were more prone to form multipolar or monopolar mitotic spindles relative to the U87MG parental cells. 25% of the scored mitosis in the U87MG EGFRvIII population were either multipolar (17%) or monopolar (8%) while none of the mitosis scored in the U87MG parental cells exhibited these phenotypes (Figure 2E). Treatment with TMZ resulted in a significant increase in mitotic death for U87MG EGFRvIII cells, but not for U87MG cells - despite a near 10-fold increase in the frequency of aberrant mitosis in these cells (Supplemental Figure 2). These observations suggest that DNA damage accumulation is i) associated with aberrant mitotic progression, and ii) associated with mitotic death in the context of EGFRvIII expression.

### BI2536 treatment inhibited PLK1 mediated phosphorylation of Rad51 Ser14, and compromised homologous recombination

**Figure 3:**
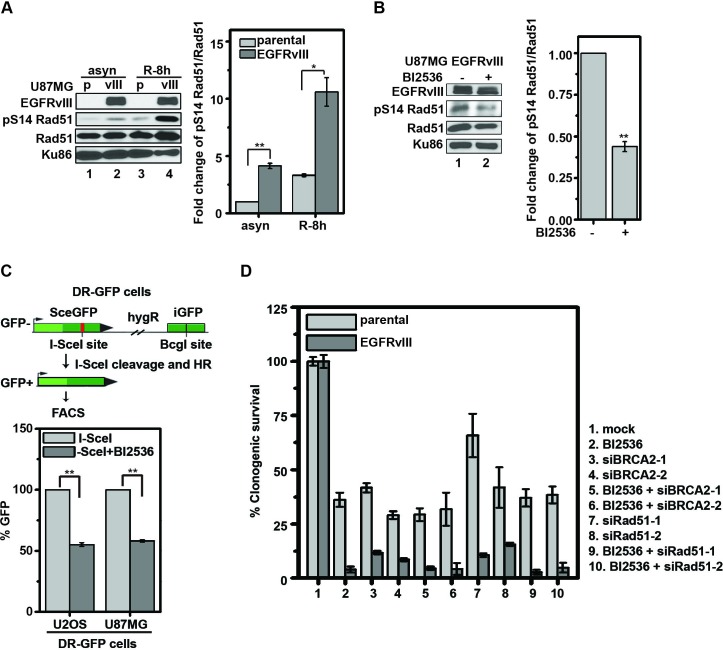
BI2536 inhibits phosphorylation of Rad51 S14 and compromises HR in glioblastoma cells **A.** (left) Representative immunoblots of U87MG parental (p) and U87MG EGFRvIII (vIII) cells for pS14 Rad51, Rad51, and Ku86. asyn, asynchronized cells; R-8 h, synchronized cells released from DTB for 8 h. (right) pS14Rad51 was normalized to the total Rad51 after correcting for protein loading using Ku86 level. **, *p* = 0.0026; *, *p* = 0.045. **B.** (left) Representative immunoblot of whole U87MG EGFRvIII cell lysate following 24 h treatment with control or 10 nM BI2536. (right) Quantitative densitometric assessment as above described. **, *p* = 0.0018.**C.** (left) Schematic summary of the DR-GFP assay. (right) Percentage of GFP-positive cells detected by FACS using U2OS DR-GFP and U87MG DR-GFP cells, separately. GFP-positive cells were scored after treatment with BI2536 (25 nM) or control for 24 h. **, *p* = 0.00056 and 0.00057 respectively. **D.** The effects of RAD51 and BRCA2 knockdown in U87MG parental and U87MG EGFRvIII cells. Cells were transfected with the various siRNAs for 24 h and re-plated overnight. BI2536 (25 nM) or control were then added. Clonogenic survivals were scored after additional 14 days. All results were shown as mean±SD.

PLK1 has been shown to phosphorylate Rad51 at serine residue 14 (pS14 Rad51) in HeLa and 293T cells to facilitate HR [26]. Given that PLK1 inhibition resulted in an increased level of DNA damage in EGFRvIII expressing glioblastoma cells, we next tested whether the PLK1-mediated Rad51 phosphorylation played a significant role in glioblastomas. The level of pS14 Rad51 was approximately 3-4-fold higher in U87MG EGFRvIII cells relative to U87MG parental cells in synchronized cell populations (Figure 3A). This level was suppressed by treatment with BI2536 (Figure 3B). BI2536 treatment also reduced the efficiency of HR by approximately 50% in the established DR-GFP assay [27] in both U87MG glioblastoma and U2OS osteosarcoma cells (Figure 3C). Taken together, these results suggest that PLK1 counteracts excessive DNA damage accumulation by promoting HR in EGFRvIII expressing glioblastoma cells. Consistent with this interpretation, siRNAs against Rad51 and BRCA2, two genes essential for HR [26], caused significantly higher toxicity in U87MG EGFRvIII cells relative to U87MG cells. Silencing Rad51 or BRCA2 did not further enhance the cytotoxic effect of BI2536 (Figure 3D and Supplemental Figure 3).

### PLK1 inhibition augments the tumoricidal effect of TMZ *in vitro* and *in vivo*

**Figure 4:**
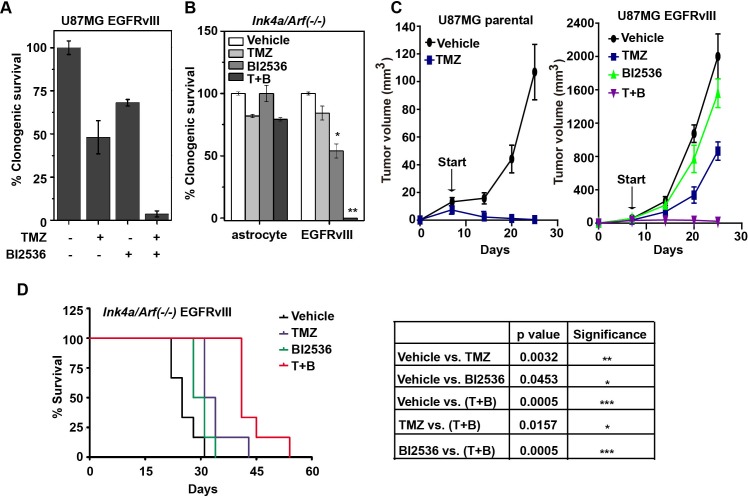
BI2536 augments the tumoricidal effect of TMZ *in vitro* and *in vitro* **A.** U87MG EGFRvIII cells were treated with TMZ (100 μM) for 24 h followed by the addition of BI2536 at 12 nM. Clonogenic survival was measured after 14 days. **B.** Murine *Ink4a/Arf(−/−)* astrocytes and derived EGFRvIII cells were treated with TMZ (50 μM, 24h) followed by BI2536 at 5 nM. Clonogenic survivals were assessed as above described. *, *p* = 0.015; **, *p* = 0.0001. **C.** Growth curve of subcutaneous U87MG parental (left) and U87MG EGFRvIII (right) xenografts in nude mice. The xenograft harboring mice were treated with control, TMZ (for 3 days starting treatment at Day 7 after tumor implantation), BI2536 (starting at Day 13), or a combination of TMZ and BI2536 (T+B) (TMZ starting at Day 7 and BI2536 starting at Day 13). **D.** (left) Survival curve of murine *Ink4a/Arf(−/−)* EGFRvIII intracranial allografts bearing mice treated with control, TMZ (starting at Day 10), BI2536 (starting at Day 13), or combination (T+B) (starting with TMZ at Day 10, then with BI2536 at Day 13). (right) p values derived from survival comparisons. 5-6 mice per group for each *in vivo* experiment. All results were shown as mean±SD. *, *p* < 0.05; **, *p* < 0.01; ***, *p* < 0.001.

Since inhibition of DDR and induction of DNA damage often result in synergistic tumor ablation [28], our results suggest that PLK1 inhibition would enhance the tumoricidal effect of TMZ, the DNA damaging chemotherapeutic agent routinely used in glioblastoma treatment. In clonogenic assays, the combination of BI2536 and TMZ treatment led to synergistic ablation of U87MG EGFRvIII cells. Exposure to 100 μM of TMZ and 12 nM of BI2536 led to approximately 50% and 30% reduction in viability, respectively. Combined treatment led to complete ablation of U87MG EGFRvIII cells (Figure 4A). Similar results were obtained using the murine *Ink4a/Arf(−/−)* and *Ink4a/Arf(−/−)* EGFRvIII cells (Figure 4B).

To confirm the validity of these results *in vivo*, we tested the combination therapy using U87MG parental and U87MG EGFRvIII in a murine subcutaneous model. Treatment was initiated 7 days after implantation, with tumor sizes < 100 mm^3^. Under these conditions, the U87MG parental xenografts were exquisitely sensitive to TMZ treatment, with complete regression after TMZ treatment (Figure 4C, left). For the U87MG EGFRvIII cells, the volumetric ratios of TMZ-treated and BI2536-treated tumors relative to vehicle treated tumors (Treatment/Control or T/C ratio, at day 25) were 64% and 79.1%, respectively. In comparison, tumors treated with both TMZ and BI2536 showed a T/C ratio of 1.9% (Figure 4C, right).

The effects of BI2536 and TMZ were further characterized in an orthotopic model where glioblastoma derived from an *Ink4a/Arf(−/−)* EGFRvIII model [29] was intracranially implanted into athymic nude mice. In this model, TMZ and BI2536 treatment prolonged the median survival of intracranial *Ink4a/Arf(−/−)* EGFRvIII tumor-bearing mice from 25 days (vehicle treated) to 32.5 days and 29.5 days, respectively. The combination further improved the median survival to 41 days (Figure 4D). Collectively, our results suggest that BI2536 augmented the tumoricidal activity of TMZ.

### Context dependency of PLK1 inhibition

**Figure 5:**
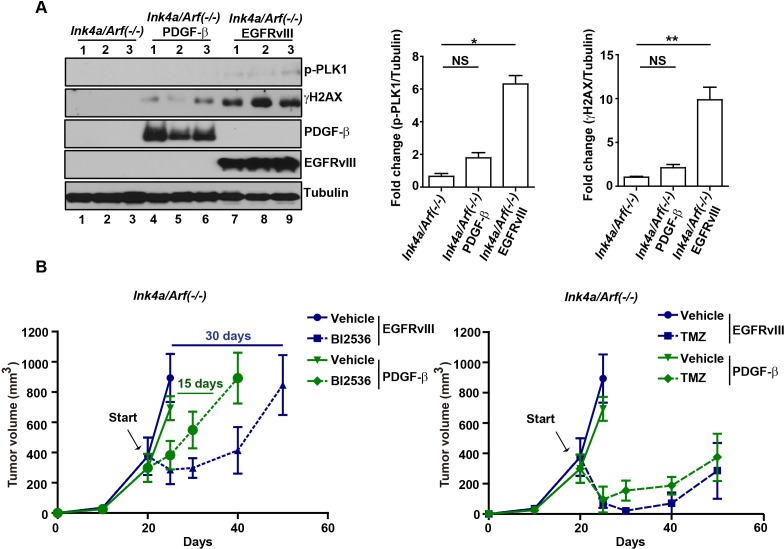
Context dependency of PLK1 inhibition **A.** (left) Enhanced p-PLK1 and γH2AX in *Ink4a/Arf(−/−)* EGFRvIII tumors. 3 representative tissue samples resected from murine *Ink4a/Arf(−/−)*, *Ink4a/Arf(−/−)* EGFRvIII or *Ink4a/Arf(−/−)* PDGF-β, separately, were lysed and analyzed by immunoblotting using antibodies against p-PLK1, γH2AX, EGFRvIII and PDGF-β. Tubulin was loaded as loading control. (right) Quantitative densitometric assessment of pT210 PLK1 or γH2AX was normalized to tubulin and fold change represent the average of 3 samples, shown as mean±SD. *, *p* < 0.05; **, *p* < 0.01. **B.** BI2536 treatment exerted greater tumoricidal effect in *Ink4a/Arf(−/−)* EGFRvIII tumors compared to *Ink4a/Arf(−/−)* PDGF-β tumors. *Ink4a/Arf(−/−)* EGFRvIII or *Ink4a/Arf(−/−)* PDGF-β cells were implanted into nu/nu mice and the treatment started when the tumor size was > 500 mm^3^. Tumor growth was monitored twice per week and represented separately to represent the comparison between BI2536 or TMZ treatment.

EGFRvIII and PDGF-β are driver oncogenes for the classical and proneural subtypes of glioblastoma, respectively [20]. Characterization of murine glioblastomas formed in the *Ink4a/Arf(−/−)* EGFRvIII [30] and *Gtv-a Ink4a/Arf(−/−)* PDGF-β model [31] revealed significantly higher levels of DNA damage accumulation in the former, as evidenced by the levels of γH2AX (Figure 5A). If the therapeutic effect of PLK1 inhibition is related to the endogenous level of DNA damage, we would predict that BI2536 would be less effective in the *Ink4a/Arf(−/−)* PDGF-β model relative to the *Ink4a/Arf(−/−)* EGFRvIII model. To test this hypothesis, glioblastoma cells derived from these models were implanted into the flank of athymic nude mice. If the mice were treated while the tumor burden was < 100 mm^3^, no significant differences in response to BI2536 were noted (Figure 4C versus Supplemental Figure 4). However, when the tumors were allowed to reach the size > 500 mm^3^ before treatment, notable difference in response to BI2536 were found between the two models, as shown in Figure 5B. BI2536 induced tumor response (as measured by tumor shrinkage and dormancy) for a period of 15 days in the *Ink4a/Arf(−/−)* PDGF-β model, after which tumors rapidly increased in size. In the *Ink4a/Arf(−/−)* EGFRvIII model, BI2536 induced tumor response for a period of 30 days (*p* < 0.05). In contrast, the anti-neoplastic effects of TMZ treatment were comparable in both models. These results suggest that the therapeutic efficacy of PLK1 inhibition is dependent on the glioblastoma genetic context and correlates with the endogenous levels of DNA damage. TMZ is a more non-selective tumor ablative agent.

### Correlation between EGFRvIII status, PLK1 expression, and DNA damage accumulation in clinical glioblastoma specimens

**Figure 6:**
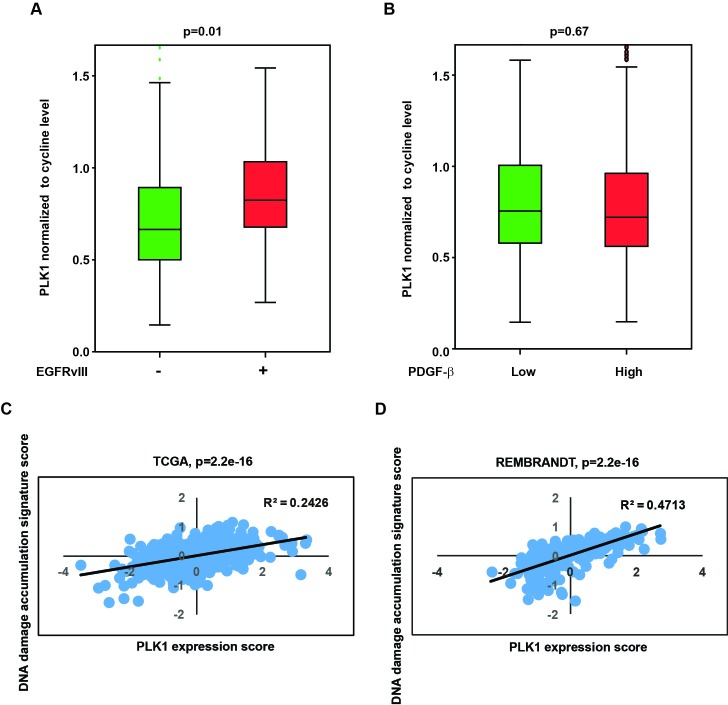
Clinical relevance of PLK1 level with EGFRvIII state and DNA damage accumulation signature **A.** Higher expression of PLK1 in EGFRvIII+ glioblastoma specimens relative to EGFRvIII- specimens. *p =* 0.01. **B.** Cyclin-normalized PLK1 expression did not correlate with PDGF-β mRNA expression. *p* = 0.67. **C.** Scatter plot showing a positive correlation between the PLK1 expression (x-axis) and DNA damage accumulation signature score (y-axis) in TCGA clinical glioblastoma dataset. **D.** Similar correlation in REMBRANDT clinical glioblastoma dataset. Linear regression was performed to generate the best-fitting line as indicated by the black line. Pearson Correlation Coefficient (R^2^) and *p*-value is as indicated.

Our results indicate that EGFRvIII expression is associated with increased PLK1 expression in glioblastoma cells (Figure 1E, 1F). We investigated further whether this association was also observed in clinical samples. Since PLK1 is expressed in a cell-cycle dependent manner, it is critical to control for cell-cycle profiles in this analysis. We utilized a previously published method and normalized PLK1 expression level to the expression of the two key mitotic cyclins, cyclin A and B [32]. A comparison using The Cancer Genome Atlas (TCGA) glioblastoma database revealed that EGFRvIII+ glioblastomas exhibited higher cyclin-normalized PLK1 expression than the EGFRvIII- glioblastomas (Figure 6A, *p* = 0.01). In contrast, cyclin-normalized PLK1 expression did not correlate with PDGF-β mRNA expression (Figure 6B, *p* = 0.67).

Our data further suggest that PLK1 expression is associated with a cell state characterized by increased DNA damage accumulation. To test this association in clinical specimens, we identified a published mRNA signature that was induced upon exposure of glioblastoma cells to ionizing radiation [33]. We used this signature as a proxy for the accumulation of DNA damage. We found that PLK1 expression correlated with the DNA damage accumulation signature in TCGA dataset (Figure 6C) and another independent glioblastoma dataset, the Repository for Molecular Brain Neoplasia Data (REMBRANDT) (Figure 6D). Despite the difference in patient population and array platform, the correlation between PLK1 mRNA expression and DNA damage accumulation signature was highly significant in both datasets (*p* < 2.2×10^−16^ for both the TCGA and REMBRANDT dataset).

### EGFR inhibitor resistant clones of glioblastoma remained uniformly sensitive to the PLK1 inhibition

**Figure 7:**
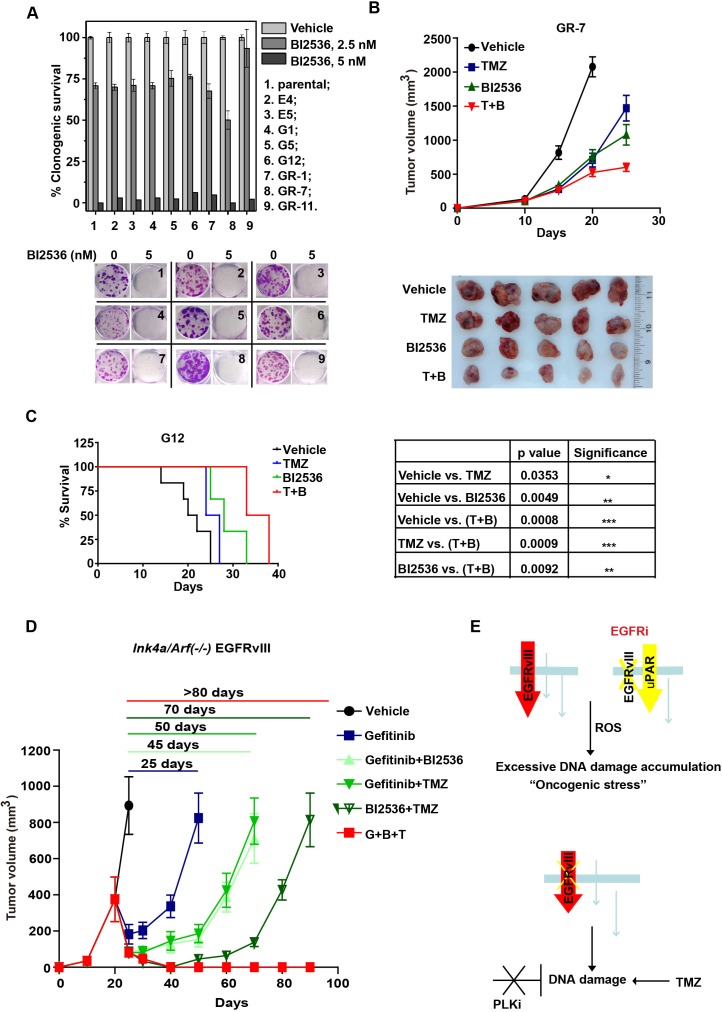
BI2536 inhibits tumor growth of EGFR inhibitor-resistant murine *Ink4a/Arf(−/−)* EGFRvIII clones and sensitizes them to the DNA damaging agent TMZ **A.** (upper) Clonogenic survival of murine *Ink4a/Arf(−/−)* EGFRvIII cells (parental) and established EGFR inhibitor resistant cells (erlotinib-resistant E4, E5 and gefitinib-resistant G1, G5, G12, GR-1, GR-7, GR-11) with BI2536 treatment. (lower) Representative colony formation images. **B.** (upper) Tumor growth curve of the subcutaneous GR-7 allografts. Nude mice bearing established GR-7 tumors in the flank were treated with control, TMZ (for 3 days starting treatment at Day 10 after implantation), BI2536 (starting at Day 13), or combined (T+B) (starting with TMZ at Day 10, then with BI2536 at Day 13). Mean tumor volume±SD are shown in 5-6 mice per group. (lower) Typical tumors isolated from each group. **C.** (left) Survival curve of intracranial G12 allografts bearing mice. The mice were treated with control, TMZ (starting at Day 10), BI2536 (starting at Day 13), or combined (T+B) (starting with TMZ at Day 10, then with BI2536 at Day 13) in 5-6 mice per group. (right) p values derived from survival comparisons. *, *p* < 0.05; **, *p* < 0.01; ***, *p* < 0.001. **D.** Tumor growth of the subcutaneous *Ink4a/Arf(−/−)* EGFRvIII allografts. Nude mice bearing established *Ink4a/Arf(−/−)* EGFRvIII tumors in the flank were treated as indicated in *Methods*. T, TMZ; B, BI2536; G, Gefitinib. Mean tumor volume±SD are shown in 5-6 mice per group. **E.** Schematic representation of “multi-orthogonal” approach. Upper panel: EGFRvIII expressing glioblastomas adapt to EGFR inhibition (EGFRi) by activation of alternative oncogenic signaling cascade, such as ones mediated by the urokinase receptor (uPAR). Other resistance mechanisms involving activation of cytoplasmic proteins, such as Src, have also been reported [42]. Despite the change in oncogenic signaling, the intrinsic physiological “architecture” of the transformed cells and the cellular dependence on DDR remain largely unaltered. Red arrow: EGFRvIII signaling. Yellow arrow: EGFR inhibition induced up-regulation of uPAR signaling. Bottom panel: As such, simultaneous inhibition of DDR and EGFR inhibition impose independent and parallel selection against glioblastoma cells. The therapeutic efficacy of the regimen is further magnified induction of additional DNA damage by temozolomide (TMZ), a DNA alkylating agent and the standard-of-care chemotherapy for glioblastomas.

We determined whether glioblastoma cells with acquired resistance to EGFR inhibitors maintain cell states with oncogenic stress that require PLK1 as a compensatory mechanism. To test this hypothesis, we characterized the effect of BI2536 on a panel of 8 murine *Ink4a/Arf(−/−)* EGFRvIII clones selected for their resistance to EGFR inhibitors. The parental *Ink4a/Arf(−/−)* EGFRvIII clone was sensitive to EGFR inhibitors. These parental cells were cultured in the presence of either Gefitinib (clones G1, G5, G12, GR-1, GR-7 and GR-11) or Erlotinib (clones E4 and E5) to select for acquired EGFR inhibitor resistance [34]. These clones utilize distinct EGFR independent-signaling pathways [34]. Remarkably, all resistant clones remain uniformly sensitive to BI2536 *in vitro* at concentrations comparable to that required to ablate the parental *Ink4a/Arf(−/−)* EGFRvIII cells (Figure 7A). This effect is specific to EGFRvIII expressing cells, as evidenced by the lack of cytotoxic effect of the BI2536 (5 nM) toward the *Ink4a/Arf(−/−)* astrocytes (Figure 1D).

The sensitivity of EGFR inhibitor resistant clones to PLK1 inhibition was further investigated using *in vivo* mice models. As shown in Figure 7B, the subcutaneous growth of GR-7 was modestly suppressed by TMZ (T/C ratio of 46.8%; Day 25) and BI2536 (T/C ratio of 34.33%; Day 25). Combined treatment significantly inhibited the growth of GR-7 with T/C ratio of 19.1% (Day 25). The effect of PLK1 inhibition on the survival of G12 intracranial tumor bearing mice was determined in Figure 7C. TMZ and BI2536 prolonged the median survival of mice bearing G12 allografts to 25.5 and 28 days, respectively, compared to 21 days in control group mice. Combined treatment prolonged the median survival to 35.5 days. These effects were near identical to those observed with the parental EGFR inhibitor-sensitive *Ink4a/Arf(−/−)* EGFRvIII cells (Figure 4D). In aggregate, these results suggest that the resistance mechanisms for PLK1 and EGFR inhibitors are fundamentally orthogonal in nature.

### Combination of Gefitinib, BI2536, and TMZ ablated formed tumors in a murine *Ink4a/Arf*(*−/−*) EGFRvIII glioblastoma model

Since our results support that Gefitinib and BI2536 exert anti-neoplastic effects in an orthogonal manner, we wished to determine whether synergy can be achieved with this combination. To this end, *Ink4a/Arf(−/−)* EGFRvIII cells were injected subcutaneously into the flank of athymic nude mice. When the average tumor volume exceeded 500 mm^3^ (see *Methods*), mice were randomized to different treatment groups (Figure 7D). Treatment with Gefitinib elicited tumor dormancy for 25 days, after which tumors became resistant and rapidly increased in size. While the addition of BI2536 lengthened the tumor response to 45 days, tumor recurrence was universal.

Since the therapeutic effect of BI2536 was enhanced by TMZ, we next determined whether the addition of TMZ to BI2536+Gefitinib further enhanced the anti-neoplastic effect of the regimen. The triple combination of Gefitinib, TMZ and BI2536 completely suppressed the tumor growth for 3 months. Such effect was not observed in any of the double combination therapies or in the *Ink4a/Arf(−/−)* PDGF-β models (Supplemental Figure 5). These results demonstrate the need for multi-orthogonal therapy and the need to tailor such therapy to the underlying molecular physiology of target cancer cell.

## DISCUSSION

With the emergence of selective inhibitors of PLK1, there has been growing interest in their clinical application to glioblastomas [35]. However, the therapeutic rationale remains poorly developed [36]. Moreover, no predictive biomarker has been identified. Through our finding of synthetic lethality between PLK1 and EGFRvIII and subsequent characterization of this genetic interaction, we provide the evidence that the anti-neoplastic effect of PLK1 inhibitors is related to the disruption of PLK1's critical roles in DNA damage response. Our results indicate that glioblastomas harboring high levels of DNA damage accumulation, including those expressing EGFRvIII, are more likely to respond to PLK1 inhibitors (Figure 4E and 4F). Further, our results suggest that *Ink4a/Arf(−/−)* EGFRvIII glioblastomas that acquired resistance to EGFR inhibition retain oncogenic stress that requiring PLK1 compensation (Figure 7E, upper panel). As such, PLK1 and EGFR inhibitor represent orthogonal therapeutic agents, with enhanced tumoricidal activity when combined. However, the glioblastoma molecular circuit is sufficiently pliant that resistant clones eventually emerge after BI2536 + Gefitinib combination therapy (Figure 5D). Complete glioblastoma ablation was achieved only with a multi-orthogonal regimen consisting of BI2536, Gefitinib, and TMZ (Figure 7E, bottom panel). Importantly, the efficacy of such regimen is influenced by the genetic context of the target glioblastoma and the associated oncogenic stress.

One important consideration pertaining to the translation of this therapeutic combination involves potential toxicity of the combination. Of note, the safety profile of each component of the combination has been well-documented. PLK1 inhibitors have been proved to be well-tolerated in phase I and II clinical trials [37] and have good brain blood barrier permeability [38]. Importantly, one PLK1 inhibitor, Volasertib (BI6727), has been advanced to phase III clinical trial testing as treatment for acute myeloid leukemia. Similarly, the clinical safety profiles of Gefitinib and TMZ are well-established for glioblastoma patients [21]. Given the observation that mice tolerated the combined therapy well and the safety profile of the individual agent, clinical translation of this therapeutic strategy warrants consideration. It is important to note that our studies were conducted in immuno-compromised murine models. As such, exploitation of innate immunity against tumor [39] may afford opportunities to decrease the number of therapeutic agents required for meaningful efficacy, thereby minimizing the risk of treatment related toxicity.

The finding that EGFR inhibitor resistant clones of glioblastoma remained sensitive to PLK1 inhibition suggests that while the resistant glioblastoma cells underwent a change in oncogenic signaling, the intrinsic physiological “architecture” of the transformed cells and the cellular dependence on DDR remained largely unaltered. From a systems perspective, it is somewhat intuitive that adopting a resistance mechanism where an alternate oncogenic mechanism is activated to drive the existing cellular circuit would be less costly than a resistance mechanism that required a complete re-alignment of fundamental cellular processes. As such, the delineation of the physiologic state of the initial tumor may lend therapeutic insights, particularly for tumors with highly pliable molecular circuits.

While it is widely appreciated that excessive DSB accumulation is a lethal phenomenon [25], the mechanisms by which DSBs trigger lethality in glioblastoma cells remain poorly understood [40]. The established functions of PLK1 [41] and the results presented here suggest prolonged cell cycle arrest and mitotic DNA damage accumulation as contributing mechanisms of DSB induced lethality. Supporting this thesis, the tumoricidal activities of PLK1 inhibition correlated with mitotic DNA damage accumulation (Figure 2C and Figure 1E). Moreover, TMZ treatment, a process that results in excessive DSB accumulation, significantly increased the frequency of aberrant mitosis and mitotic death (Figure 2E).

In summary, our study demonstrated pre-clinical efficacy of combining TMZ, PLK1 and EGFR inhibitors as treatment for EGFRvIII expressing glioblastomas. More broadly speaking, the study outlines the rationale for a “multi-orthogonal” approach integrating conventional chemotherapy with inhibitors directed against different forms of oncogenes and oncogenic stress (Figure 5F). Importantly, the “multi-orthogonal” approach needs to be tailored to the molecular physiology of the target cancer. With an ever-expanding catalogue of the mutational landscape of glioblastomas, dedication to understanding the molecular physiologies associated with these landscapes will be essential for multi-orthogonal based therapeutic approaches. Based on the present study, it is our belief that optimal oncologic strategies against cancers with highly pliable molecular circuit will require meaningful integration of orthogonally acting, targeted agents and non-selective chemotherapeutic agent tailored to the genetic landscape of the tumor.

## MATERIALS AND METHODS

### Cell lines and reagents

The U87MG parental, U87MG EGFRvIII, U178MG tet-EGFRvIII cell lines, and transformed mouse *Ink4a/Arf(−/−)* astrocytes, *Ink4a/Arf(−/−)* EGFRvIII cells, *Ink4a/Arf(−/−)* EGFRvIII Gefitinib/Erlotinib resistant cells, and *Ink4a/Arf(−/−)* PDGF-β cells have been previously described [29, 31, 42]. U87MG parental H2B-GFP and U87MG EGFRvIII H2B-GFP cells were constructed by infecting U87MG parental and U87MG EGFRvIII cells with a H2B-GFP construct [43] generously provided by Dr. David Pellman (Dana Farber Cancer Institute, Boston). TMZ (AK Scientific, Mountain View, CA) and BI2536 (ChemieTek, Indianapolis, IN) were dissolved in DMSO (Sigma Aldrich, St. Louis, MO). Doxycycline (Clontech, Mountain View, CA) was dissolved in deionized water. Cells were cultured in DMEM medium (Gibco) supplemented with 10% fetal bovine serum (FBS, Gibco), 1% Pen-Strep (Gibco) and 1% GlutaMax (Gibco) unless otherwise specified. Tetracycline free serum (Clontech, Mountain View, CA) was used for experiments involving doxycycline.

### siRNA library screen

The initial siRNA screen was performed with the DNA damage response subset v2.0 library (Qiagen, Valencia, CA) as previously described [11]. Each gene target is represented by two distinct siRNAs as previously described [44]. In brief, The QIAGEN siRNA library was grided into 96 well plates. Each plate also contained 2 GFP-targeted siRNAs, 2 LacZ-targeted siRNAs at 20 nM. For each plate, there were 16 wells containing no siRNA as controls. U87 or U87-EGFRvIII cells were seeded in 96-well plates (BD Biosciences) at 1,000 cells per well in 80 ul of medium. Twenty-four hours later, a transfection mix of 15.5 ml of OptiMEM (Invitrogen), 0.5 ml of HiPerFect (QIAGEN), and 4 ml of 2 mM siRNA oligonucleotide was added to each well. Viability at 96 hours was measured using the CellTiter-Glo Luminescent Cell Viability Assay kit (Promega). The experiment was performed twice to allow statistical analysis of the targets. The corrected viability for each siRNA oligonucleotide was calculated as a percentage of the mean viability of the 16 control wells on each plate. The corrected viability of the U87MG-EGFRvIII cell line was divided by the corrected viability of the U87MG cell line to calculate the relative viability for each respective gene target. The mean viability of the U87MG-EGFRvIII relative to the U87MG cell line for each gene target, along with the SEM, was calculated from 4 individual corrected viability values that represent duplicate results from the 2 different oligonucleotides on each plate targeting a particular gene. The siRNA targets are then ranked based on this index [28].

### Live cell imaging

U87MG H2B-GFP and U87MG EGFRvIII H2B-GFP cells were grown on 12-well glass-bottom dishes (MatTek, Ashland, MA) overnight (5×10^4^ cells per well). Images were acquired automatically from each well using a Nikon TE2000E PFS inverted microscope fitted with a 20×Nikon Plan Fluor objective (Nikon, Melville, NY), a linearly encoded stage (Prior ProScan, Prior Scientific, Rockland, MA) and a Hamamatsu Orca-ER CCD camera (Hamamatsu, Bridgewater, NJ). The microscope was controlled using NIS Element (Nikon). The microscope was housed in a custom-designed 37°C chamber with a secondary internal chamber that delivered humidified 5% CO_2_. Fluorescence and differential interference contrast images were obtained every 15 min for a period of 72 h. For experiments involving TMZ, the cells were treated with 100 μM TMZ for 24 h prior to imaging.

### I-SceI recombination assay

DR-GFP assays were performed using U87MG DR-GFP and U2OS DR-GFP subclones as previously described [27]. Briefly, plasmids phprtDRGFP and pCBASce were generously provided by Dr. David Weinstock (Dana Farber Cancer Institute, Boston). U87MG and U2OS were transfected with phprtDRGFP via FuGENE HD (Roche, Indianapolis, IN). Subclones harboring single stable integration identified by Southern blotting were generously provided by Dr. David Kozono (Dana Farber Cancer Institute, Boston). 24 h after pCBASce transfection to induce DSB at I-SceI site of the integrated phprtDRGFP construct, cells were treated with either BI2536 (25 nM) or control for an additional 24 h, then trypsinized and subjected to FACS analysis to identify the proportion of GFP-expressing cells.

### Subcutaneous xenograft and GEMM-derived glioblastoma models

All animal studies were performed in accordance with the Animal Care and Use Rules at the University of California San Diego under protocol S13070. 1×10^6^ cells of exponentially expanding U87MG, U87MG EGFRvIII, murine *Ink4a/Arf(−/−)* EGFRvIII, its gefitinib resistant line GR-7, or murine *Ink4a/Arf(−/−)* PDGF-β, in 100 μL of PBS were injected into the right flank of 4-5weeks old athymic nude mice. Tumors were measured with a vernier caliper, and tumor volumes(TVs) were calculated using width (a) and length (b) measurements (TV = a^2^×b/2, where a≤b). Relative tumor volume (RTV) was calculated by (RTV = TV_t_/TV_0_, where TV_0_ is the tumor volume measured when starting drug treatment). The anti-tumor effect of drug treatment was calculated by drug treated/control (T/C) ratio (T/C = RTV_treated_÷RTV_control_×100%). Mice were euthanized when tumor volume reached 2000 mm^3^ or tumors became ulcerated in accordance with our institutional guidelines for animal welfare and experimental conduct.

### Intracranial brain tumor allograft models

10^5^ cells of the exponentially expanding murine *Ink4a/Arf(−/−)* EGFRvIII or G12 cells were injected into 4-5weeks old athymic nude mice using a mouse stereotaxic instrument (Stoelting Co, Wood Dale, IL) according to the protocol previously described [45]. Survival was recorded until the onset of neurologic sequelae or cachexia. Mice were euthanized in accordance with our institutional guidelines for animal welfare and experimental conduct. The survival curve was calculated by GraphPad Prism 5 (GraphPad, La Jolla, CA) using the method of Kaplan-Meier. p values were analyzed using the logrank (Mantel-Cox) test.

TMZ was given at 15 mg/kg by oral gavage once per day for three days at indicated time point after implantation. BI2536 was given consecutively at 25 mg/kg by i.v. injection twice per week for four weeks beginning at indicated time point. Gefitinib was administered via oral gavage at 200 mg/kg weight once per day, 5 days per week for four weeks. For all experiments, 5-6 mice were randomized to each treatment group.

### Validation of screen results using clonogenic assays

Confirmation of PLK1 results was performed using two additional siRNAs directed targeting PLK1 that were distinct from the siRNAs included in the library (Hs_PLK1_6 FlexiTube siRNA and Hs_PLK1_7 FlexiTube siRNA, Qiagen). 20 nM of siRNA was transfected using RNAiMax (Invitrogen, Carlsbad, CA) according to manufacturer's protocol for 48 h prior to subsequent Western blotting and clonogenic assay. Cells were re-plated in serial dilution for clonogenic survival assessment in triplicates and repeated at least twice as previously described [11].

### Cell synchronization by double thymidine blocking (DTB)

Briefly, the seeded cells were washed with phosphate-buffered saline (PBS, Invitrogen) then incubated with 2 mM thymidine (Sigma) for 16 h. Fresh complete DMEM media was incubated for 8 h to release cells. Then the cells were subjected to a second thymidine block by thymidine for another 16 h. The cells were released to fresh DMEM media. Cell cycle profiling was analyzed using FACS Calibur system (BD Biosciences, San Jose, CA) as previously described [46].

### Western blot

Lysates were prepared using a RIPA lysis buffer (Sigma) supplemented with a cocktail of protease inhibitors (Roche, Indianapolis, IN), incubated for 15 min on ice, and then clarified by centrifugation. Equal amounts of protein were resolved by SDS-polyacrylamide gel electrophoresis and electro-transferred to nitrocellulose membranes (Invitrogen, Carlsbad, CA). Membranes were blocked for 1 h in 5% fat-free milk dissolved in TBS containing 0.1% Tween-20 and incubated overnight at 4°C with indicated antibodies, including anti-EGFR (Cell Signaling Tech [CST], Danvers, MA), anti-pT210 PLK1 (CST), anti-pSer10 Histone H3 (labeled as pH3, a marker of cells in mitosis (CST), anti-γH2AX (a marker for DNA damage, Millipore, Billerica, MA) and anti-pS14 Rad51 (kindly provided by Dr. Fumiko Esashi, University of Oxford, UK), diluted in the same blocking buffer. For loading control, membranes were probed with anti-β-actin (Sigma) or anti-Ku86 (Santa Cruz Biotech, Dallas, TX). After washing, membranes were incubated with appropriate secondary (Pierce, Rockford, IL) antibodies conjugated to horseradish peroxidase. For total protein level of PLK1 and Rad51, the p-antibody probed membranes were stripped and re-probed with anti-PLK1 (Santa Cruz Biotech) or anti-Rad51 (Santa Cruz Biotech) antibodies, respectively. Blots were developed with SuperSignal Chemiluminescence reagent (Pierce) and scanned with Perfection V700 photo scanner (Epson). The band density was analyzed by AlphaView (ProteinSimple). Phosphorylation level of Rad51 and PLK1 was compared with its total protein level, respectively, after normalized with loading control individually.

### Comet assay

The comet assay (Trevigen, Gaithersburg, MD) was performed according to manufacturer's protocol using neutral conditions. After lysis, the slides were washed twice with 1× Tris-borate EDTA buffer solution, pH 8.3 (TBE) for 10 min. The slides were placed in a horizontal electrophoresis chamber and covered with TBE buffer. Electrophoresis was carried out at the rate of 1.0 V/cm for 20 min. The slides were removed from the electrophoresis chamber, washed in deionized water for 5 min and immersed in ice cold 100% ethanol for 5 min. Subsequently, the slides were air dried, DNA was stained with 50 μl of SYBR Green I dye (Trevigen, 1:10,000 in Tris-EDTA buffer, pH 7.5) for 20 min in the refrigerator and immediately analyzed using upright fluorescence microscope (Nikon, Melville, NY), and data was analyzed using CometScore (TriTek, Sumerduck, VA).

### Immunofluorescence staining

After drug treatment, cells were trypsinized, washed once with PBS, centrifuged at 500 rpm for 5 min using a cytospin (Thermo Fisher Scientific, Waltham, MA) and seeded onto poly-l-lysine (PLL)-coated coverslips. The cells were then fixed with 4% para-formaldehyde for 20 min, blocked in 2% BSA/PBS for 30 min, and incubated in primary anti-pH3 and anti-γH2AX antibody overnight (4°C). Cells were then washed three times in 2% BSA/PBS, incubated in Alexa Fluor 488 and Alexa Fluor 594 secondary antibodies for 1 hour at room temperature. DAPI was added to stain nuclei. Cells were imaged on an upright fluorescence microscope (Nikon, Melville, NY), and the data was analyzed using FociCounter (Anna Jucha, University of Wrocław, Poland). Cells were scored based on whether they harbor ≥10 γH2AX foci.

For multipolar and monopolar mitotic spindles staining, cells were washed with PBS and fixed with cold methanol for 20 min at −20°C, followed by incubation with primary antibodiesmin including Cep192 (SPD-2, A. Dammermann, K. Oegema Lab) and α-tubulin (DM1α, Sigma). Images were recorded on a Deltavision microscope at 1 × 1 binning with a 100× NA 1.3 U-planApo objective. Z-stacks (0.2 μm sections) were deconvolved using softWorRx (Applied Precision) and maximum intensity projections were imported into Adobe Photoshop CS4 (Adobe) for analysis.

### Flow cytometry

After fixation, cells were permeablized by PBS containing 0.25% Triton X-100 for 15 min followed by incubation with primary antibodies against pH3 (CST), and γH2AX (Millipore) for 1h. After wash once with PBS containing 1% BSA, cells were incubated with Alexa Fluor 488-conjugated goat anti-rabbit IgG (H+L) and Alexa Fluor 647-conjugated goat anti-mouse IgG1 antibodies (Invitrogen) for 30 min. The cells were then treated with propidium iodide (PI)/RNase A staining buffer for another 30 min. Fluorescence-activated cell sorting (FACS) was performed with FACS Calibur (BD Biosciences, San Jose, CA). For negative control, the samples were incubated with secondary antibodies (without incubation with the primary antibodies) and PI/RNase A staining buffer. 3×10^4^ cells were analyzed for each sample. Data was analyzed with FlowJo software (BD Bioscience).

### Cell viability assays

Cells were seeded in a 96-well plate at a density of 1×10^3^ cells per well. 12-15 h later, drugs or control were added to achieve indicated concentration in quadruplicate wells and incubated for 72 h. Viability was assessed with the WST-1 cell proliferation reagent according to the manufacturer's instructions (Clontech, Mountain View, CA). Drug treatments were normalized to the control cells for each cell line to calculate percent cell viability.

Clonogenic survival assays were performed as previously described [11].

### Analysis the TCGA and REMBRANDT glioblastoma datasets

The EGFRvIII status of the TCGA glioblastoma samples were provided by Dr. Cameron Brennan (Memorial Sloan Kettering Cancer Center, New York). TCGA mRNA expression data acquired via Affymetrix HT Human Genome U133 array were downloaded as Level 3 gene collapsed data (https://tcga-data.nci.nih.gov/tcga/). PLK1 mRNA expression data was normalized to cyclin A and B enrichment scores (ES) using single sample Gene Set Enrichment Analysis (ssGSEA) employing previously published methods [47] and compared by EGFR or PDGF-β status. A box and whiskers plot was employed. p values were obtained by boot strapping the individual test statistics from two sided *t*-tests against the test statistics from 1500 simulations using random gene lists.

Genomic expression sets and clinical data were acquired from the TCGA Data Portal (May 2013) https://tcga-data.nci.nih.gov/tcga/ and the REMBRANDT https://caintegrator.nci.nih.gov/rembrandt/. A published DNA damage accumulation mRNA signature that was induced by IR in glioblastoma cells was identified [33], then correlated the signature to PLK1 expression. Values of probes designed to assess the same gene were averaged. Expression sets were generated using the Bioconductor package http://www.bioconductor.org/using the statistics software R http://www.r-project.org/ (Mountain View, CA).

### Statistical analysis

In general, data were presented as the means with their respective standard errors (SEM) or standard deviation (SD). Significance was tested by unpaired two-tailed Student *t* test using Office Excel 2007 (MicroSoft, Santa Clara, CA) unless otherwise indicated. *p* values < 0.05 were considered statistically significant. Details for additional methods, including double thymidine blocking (DTB) synchronization, flow cytometry, Western blotting, Comet assay, Immunofluorescence staining, Clinical analysis, Cell viability assay, are described in SI text.

## SUPPLEMENTARY MATERIALS FIGURES



## References

[1] Haigis KM, Sweet-Cordero A (2011). New insights into oncogenic stress. Nat Genet.

[2] Malumbres M (2011). Oncogene-induced mitotic stress: p53 and pRb get mad too. Cancer cell.

[3] Bartek J, Ng K, Bartek J, Fischer W, Carter B, Chen CC (2012). Key concepts in glioblastoma therapy. J Neurol Neurosurg Psychiatry.

[4] Luo J, Solimini NL, Elledge SJ (2009). Principles of cancer therapy: oncogene and non-oncogene addiction. Cell.

[5] Luo J, Emanuele MJ, Li D, Creighton CJ, Schlabach MR, Westbrook TF, Wong KK, Elledge SJ (2009). A genome-wide RNAi screen identifies multiple synthetic lethal interactions with the Ras oncogene. Cell.

[6] Chan DA, Giaccia AJ (2011). Harnessing synthetic lethal interactions in anticancer drug discovery. Nat Rev Drug Discov.

[7] Shen WH, Balajee AS, Wang J, Wu H, Eng C, Pandolfi PP, Yin Y (2007). Essential role for nuclear PTEN in maintaining chromosomal integrity. Cell.

[8] Halazonetis TD, Gorgoulis VG, Bartek J (2008). An oncogene-induced DNA damage model for cancer development. Science.

[9] Bartkova J, Horejsi Z, Koed K, Kramer A, Tort F, Zieger K, Guldberg P, Sehested M, Nesland JM, Lukas C, Orntoft T, Lukas J, Bartek J (2005). DNA damage response as a candidate anti-cancer barrier in early human tumorigenesis. Nature.

[10] Mendes-Pereira AM, Martin SA, Brough R, McCarthy A, Taylor JR, Kim JS, Waldman T, Lord CJ, Ashworth A (2009). Synthetic lethal targeting of PTEN mutant cells with PARP inhibitors. EMBO Mol Med.

[11] Nitta M, Kozono D, Kennedy R, Stommel J, Ng K, Zinn PO, Kushwaha D, Kesari S, Inda MM, Wykosky J, Furnari F, Hoadley KA, Chin L, DePinho RA, Cavenee WK, D'Andrea A (2010). Targeting EGFR induced oxidative stress by PARP1 inhibition in glioblastoma therapy. PLoS One.

[12] Stommel JM, Kimmelman AC, Ying H, Nabioullin R, Ponugoti AH, Wiedemeyer R, Stegh AH, Bradner JE, Ligon KL, Brennan C, Chin L, DePinho RA (2007). Coactivation of receptor tyrosine kinases affects the response of tumor cells to targeted therapies. Science.

[13] Vivanco I, Robins HI, Rohle D, Campos C, Grommes C, Nghiemphu PL, Kubek S, Oldrini B, Chheda MG, Yannuzzi N, Tao H, Zhu S, Iwanami A, Kuga D, Dang J, Pedraza A (2012). Differential sensitivity of glioma- versus lung cancer-specific EGFR mutations to EGFR kinase inhibitors. Cancer Discov.

[14] Zhang N, Wu X, Yang L, Xiao F, Zhang H, Zhou A, Huang Z, Huang S (2012). FoxM1 inhibition sensitizes resistant glioblastoma cells to temozolomide by downregulating the expression of DNA-repair gene Rad51. Clin Cancer Res.

[15] Huang HS, Nagane M, Klingbeil CK, Lin H, Nishikawa R, Ji XD, Huang CM, Gill GN, Wiley HS, Cavenee WK (1997). The enhanced tumorigenic activity of a mutant epidermal growth factor receptor common in human cancers is mediated by threshold levels of constitutive tyrosine phosphorylation and unattenuated signaling. J Biol Chem.

[16] Brennan CW, Verhaak RG, McKenna A, Campos B, Noushmehr H, Salama SR, Zheng S, Chakravarty D, Sanborn JZ, Berman SH, Beroukhim R, Bernard B, Wu CJ, Genovese G, Shmulevich I, Barnholtz-Sloan J (2013). The somatic genomic landscape of glioblastoma. Cell.

[17] Gan HK, Cvrljevic AN, Johns TG (2013). The epidermal growth factor receptor variant III (EGFRvIII): where wild things are altered. FEBS J.

[18] Kastenhuber ER, Huse JT, Berman SH, Pedraza A, Zhang J, Suehara Y, Viale A, Cavatore M, Heguy A, Szerlip N, Ladanyi M, Brennan CW (2014). Quantitative assessment of intragenic receptor tyrosine kinase deletions in primary glioblastomas: their prevalence and molecular correlates. Acta Neuropathol.

[19] Louis DN, Ohgaki H, Wiestler OD, Cavenee WK, Burger PC, Jouvet A, Scheithauer BW, Kleihues P (2007). The 2007 WHO classification of tumours of the central nervous system. Acta Neuropathol.

[20] Ng K, Kim R, Kesari S, Carter B, Chen CC (2012). Genomic profiling of glioblastoma: convergence of fundamental biologic tenets and novel insights. J Neurooncol.

[21] Mellinghoff IK, Wang MY, Vivanco I, Haas-Kogan DA, Zhu S, Dia EQ, Lu KV, Yoshimoto K, Huang JH, Chute DJ, Riggs BL, Horvath S, Liau LM, Cavenee WK, Rao PN, Beroukhim R (2005). Molecular determinants of the response of glioblastomas to EGFR kinase inhibitors. N Engl J Med.

[22] Tanaka K, Babic I, Nathanson D, Akhavan D, Guo D, Gini B, Dang J, Zhu S, Yang H, De Jesus J, Amzajerdi AN, Zhang Y, Dibble CC, Dan H, Rinkenbaugh A, Yong WH (2011). Oncogenic EGFR signaling activates an mTORC2-NF-kappaB pathway that promotes chemotherapy resistance. Cancer Discov.

[23] Inda MM, Bonavia R, Mukasa A, Narita Y, Sah DW, Vandenberg S, Brennan C, Johns TG, Bachoo R, Hadwiger P, Tan P, Depinho RA, Cavenee W, Furnari F (2010). Tumor heterogeneity is an active process maintained by a mutant EGFR-induced cytokine circuit in glioblastoma. Genes Dev.

[24] Paulsen RD, Soni DV, Wollman R, Hahn AT, Yee MC, Guan A, Hesley JA, Miller SC, Cromwell EF, Solow-Cordero DE, Meyer T, Cimprich KA (2009). A genome-wide siRNA screen reveals diverse cellular processes and pathways that mediate genome stability. Molecular cell.

[25] Bahassi eM (2011). Polo-like kinases and DNA damage checkpoint: beyond the traditional mitotic functions. Exp Biol Med (Maywood).

[26] Yata K, Lloyd J, Maslen S, Bleuyard JY, Skehel M, Smerdon SJ, Esashi F (2012). Plk1 and CK2 act in concert to regulate Rad51 during DNA double strand break repair. Molecular cell.

[27] Weinstock DM, Nakanishi K, Helgadottir HR, Jasin M (2006). Assaying double-strand break repair pathway choice in mammalian cells using a targeted endonuclease or the RAG recombinase. Methods Enzymol.

[28] Chen CC, Kennedy RD, Sidi S, Look AT, D'Andrea A (2009). CHK1 inhibition as a strategy for targeting Fanconi Anemia (FA) DNA repair pathway deficient tumors. Mol Cancer.

[29] Bachoo RM, Maher EA, Ligon KL, Sharpless NE, Chan SS, You MJ, Tang Y, DeFrances J, Stover E, Weissleder R, Rowitch DH, Louis DN, DePinho RA (2002). Epidermal growth factor receptor and Ink4a/Arf: convergent mechanisms governing terminal differentiation and transformation along the neural stem cell to astrocyte axis. Cancer Cell.

[30] Zhu H, Acquaviva J, Ramachandran P, Boskovitz A, Woolfenden S, Pfannl R, Bronson RT, Chen JW, Weissleder R, Housman DE, Charest A (2009). Oncogenic EGFR signaling cooperates with loss of tumor suppressor gene functions in gliomagenesis. Proc Natl Acad Sci U S A.

[31] Hambardzumyan D, Amankulor NM, Helmy KY, Becher OJ, Holland EC (2009). Modeling Adult Gliomas Using RCAS/t-va Technology. Transl Oncol.

[32] Kao WH, Riker AI, Kushwaha DS, Ng K, Enkemann SA, Jove R, Buettner R, Zinn PO, Sanchez NP, Villa JL, D'Andrea AD, Sanchez JL, Kennedy RD, Chen CC, Matta JL (2011). Upregulation of Fanconi anemia DNA repair genes in melanoma compared with non-melanoma skin cancer. J Invest Dermatol.

[33] Otomo T, Hishii M, Arai H, Sato K, Sasai K (2004). Microarray analysis of temporal gene responses to ionizing radiation in two glioblastoma cell lines: up-regulation of DNA repair genes. J Radiat Res.

[34] Wykosky J, Hu J, Gomez G, Taylor T, Villa GR, Pizzo D, Vandenberg S, Thorne AH, Chen CC, Mischel PS, Gonias SL, Furnari F (2014). Activation of Urokinase Receptor Signaling and Inhibition of Bim Expression Induces Resistance to EGF Receptor-targeting Drugs in Mutant EGFR Glioblastoma. Cancer Res (in press).

[35] Steegmaier M, Hoffmann M, Baum A, Lenart P, Petronczki M, Krssak M, Gurtler U, Garin-Chesa P, Lieb S, Quant J, Grauert M, Adolf GR, Kraut N, Peters JM, Rettig WJ (2007). BI 2536, a potent and selective inhibitor of polo-like kinase 1, inhibits tumor growth *in vivo*. Curr Biol.

[36] Szczurek E, Misra N, Vingron M (2013). Synthetic sickness or lethality points at candidate combination therapy targets in glioblastoma. Int J Cancer.

[37] Mross K, Frost A, Steinbild S, Hedbom S, Rentschler J, Kaiser R, Rouyrre N, Trommeshauser D, Hoesl CE, Munzert G (2008). Phase I dose escalation and pharmacokinetic study of BI 2536, a novel Polo-like kinase 1 inhibitor, in patients with advanced solid tumors. J Clin Oncol.

[38] Danovi D, Folarin A, Gogolok S, Ender C, Elbatsh AM, Engstrom PG, Stricker SH, Gagrica S, Georgian A, Yu D, U KP, Harvey KJ, Ferretti P, Paddison PJ, Preston JE, Abbott NJ (2013). A high-content small molecule screen identifies sensitivity of glioblastoma stem cells to inhibition of polo-like kinase 1. PloS one.

[39] Liu Y, Zeng G (2012). Cancer and innate immune system interactions: translational potentials for cancer immunotherapy. J Immunother.

[40] Kesari S, Advani SJ, Lawson JD, Kahle KT, Ng K, Carter B, Chen CC (2011). DNA damage response and repair: insights into strategies for radiation sensitization of gliomas. Future oncology (London, England).

[41] Vitale I, Galluzzi L, Castedo M, Kroemer G (2011). Mitotic catastrophe: a mechanism for avoiding genomic instability. Nature reviews.

[42] Fenton TR, Nathanson D, Ponte de Albuquerque C, Kuga D, Iwanami A, Dang J, Yang H, Tanaka K, Oba-Shinjo SM, Uno M, Inda MM, Wykosky J, Bachoo RM, James CD, DePinho RA, Vandenberg SR (2012). Resistance to EGF receptor inhibitors in glioblastoma mediated by phosphorylation of the PTEN tumor suppressor at tyrosine 240. Proc Natl Acad Sci U S A.

[43] Ganem NJ, Godinho SA, Pellman D (2009). A mechanism linking extra centrosomes to chromosomal instability. Nature.

[44] Kennedy RD, Chen CC, Stuckert P, Archila EM, De la Vega MA, Moreau LA, Shimamura A, D'Andrea AD (2007). Fanconi anemia pathway-deficient tumor cells are hypersensitive to inhibition of ataxia telangiectasia mutated. J Clin Invest.

[45] Li J, Zhu S, Kozono D, Ng K, Futalan D, Shen Y, Akers JC, Steed T, Kushwaha D, Schlabach M, Carter BS, Kwon CH, Furnari F, Cavenee W, Elledge S, Chen CC (2014). Genome-wide shRNA screen revealed integrated mitogenic signaling between dopamine receptor D2 (DRD2) and epidermal growth factor receptor (EGFR) in glioblastoma. Oncotarget.

[46] Whitfield ML, Zheng LX, Baldwin A, Ohta T, Hurt MM, Marzluff WF (2000). Stem-loop binding protein, the protein that binds the 3′ end of histone mRNA, is cell cycle regulated by both translational and posttranslational mechanisms. Molecular and cellular biology.

[47] Barbie DA, Tamayo P, Boehm JS, Kim SY, Moody SE, Dunn IF, Schinzel AC, Sandy P, Meylan E, Scholl C, Frohling S, Chan EM, Sos ML, Michel K, Mermel C, Silver SJ (2009). Systematic RNA interference reveals that oncogenic KRAS-driven cancers require TBK1. Nature.

