# Supplementary material for: Orthogonal targeting of EGFRvIII expressing glioblastomas through simultaneous EGFR and PLK1 inhibition
Source: Oncotarget. 2015 May 5;6(14):11751–67. doi: 10.18632/oncotarget.3996 (PMC4494902; doi:10.18632/oncotarget.3996)
Supplement: Supplementary file 1 [file oncotarget-06-11751-s001.pdf]

Orthogonal targeting of EGFRvIII expressing glioblastomas through simultaneous EGFR and PLK1 inhibition

Supplementary Materials

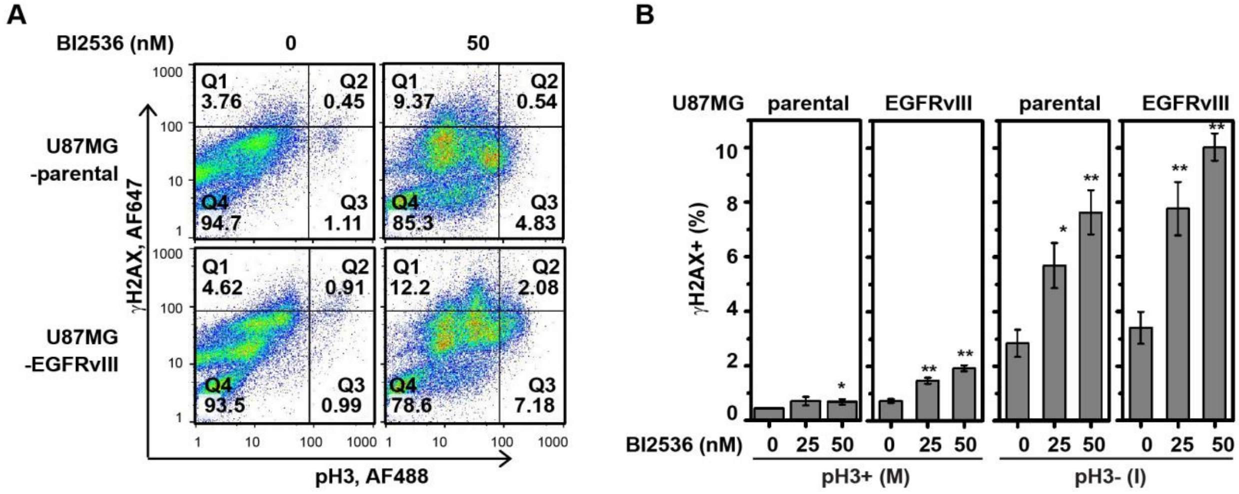

Supplemental Figure 1: BI2536 treatment increased the accumulation of γH2AX+/ pH3+ cells in U87MG EGFRvIII cells relative to U87MG parental cells. **A.** Representative FACS images of vehicle and BI2536 treated cells (50 nM for 24 h). **B.** Quantitation of cell populations with γH2AX+/pH3+ and γH2AX+/pH3- staining. Results were shown as mean±SEM. *, p=0.026, and 0.025; **, p=0.0031 for BI2536 treated samples respect to vehicle control in U87MG parental cells respectively; **, p=0.0022, 0.0007, 0.0035, 0.0099 for BI2536 treated samples respect to vehicle control in U87MG EGFRvIII cells respectively.

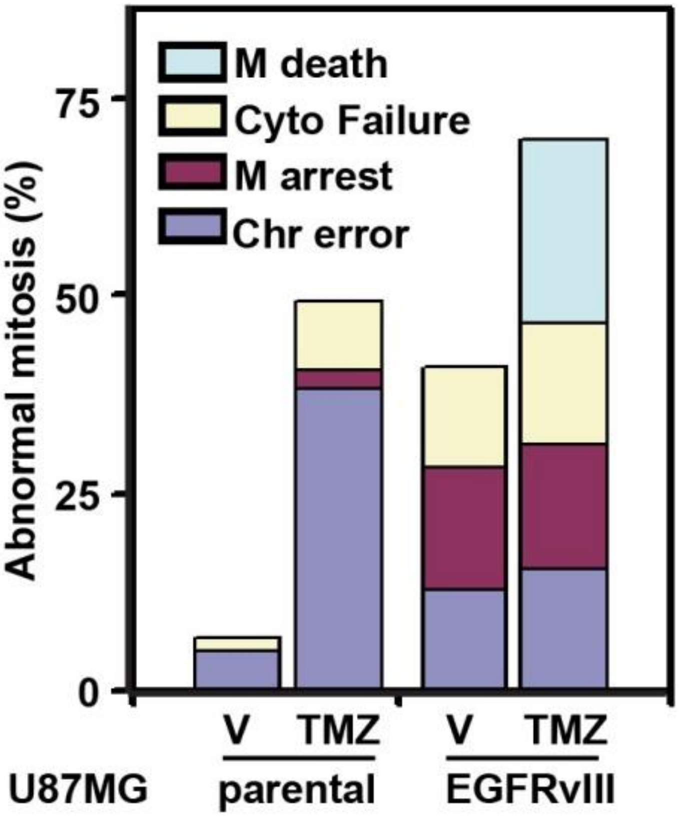

Supplemental Figure 2: Frequencies of aberrant mitotic events in U87MG parental and U87MG EGFRvIII cells after TMZ treatment. The H2B-GFP harboring cells were monitored from 24 h after TMZ (100μM) treatment. Serial images were taken every 15 min. Aberrant mitosis was classified into mitotic (M) death, cytokinesis failure (Cyto Fail), mitotic arrest, and chromosome segregation error (Chr error). Approximately 100 cells in each condition were scored. Results were shown as mean±SD.

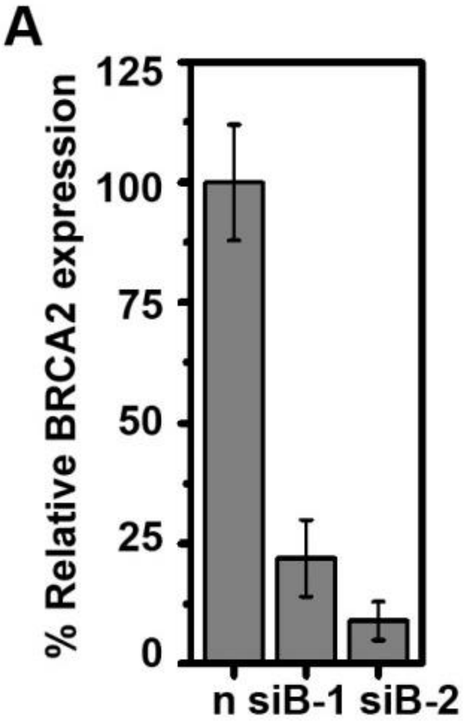

Supplemental Figure 3: BRCA2 and Rad51 were knocked down by siBRCA2s and siRad51s respectively in U87MG EGFRvIII cells. **A.** Relative BRCA2 expression normalized to GAPDH with transfection of siBRCA2-1 (siB-1) and siBRCA2-2 (siB-2) at 20 nM for 48 h. **B.** Relative Rad51 expression normalized to GAPDH with transfection of siRad51-1 (siR-1) and siRad51-2 (siR-2) at 20 nM for 48 h. Results were shown as mean±SD.

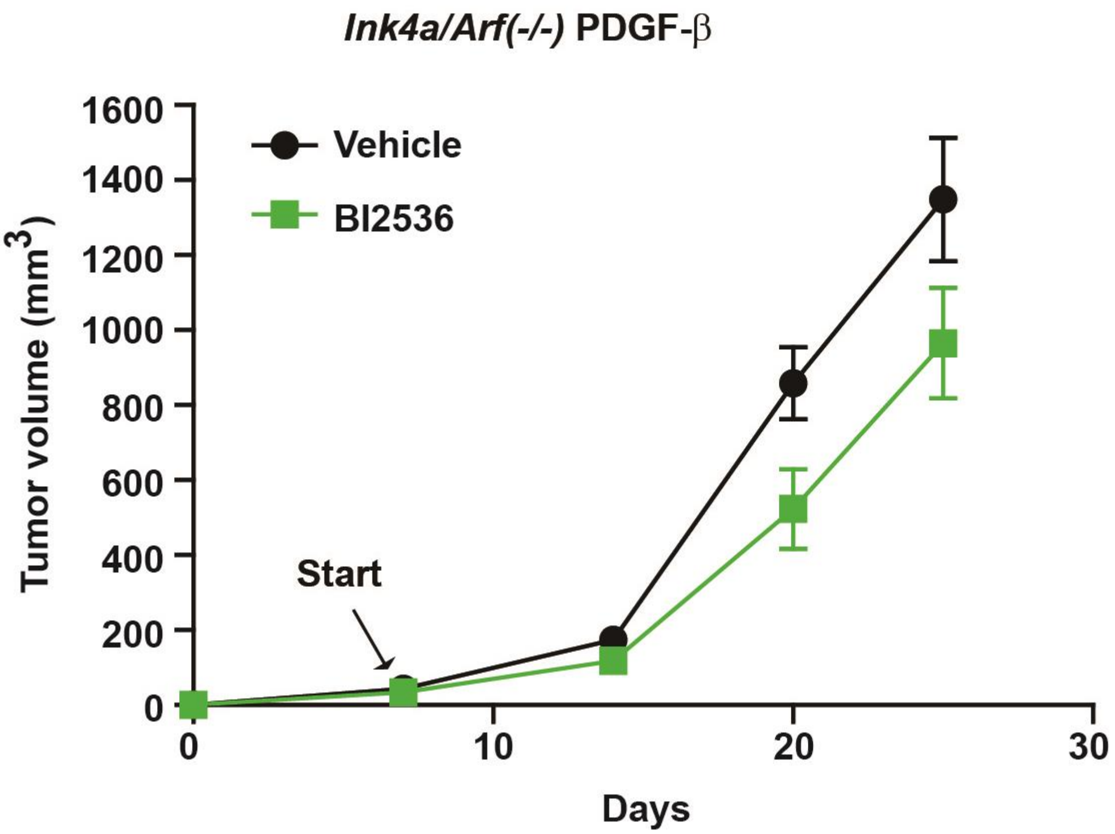

Supplemental Figure 4: Tumor growth curve of the subcutaneous *Ink4a/Arf(-/-)* PDGF-β GEMM-derived mouse models. Nude mice bearing established *Ink4a/Arf(-/-)* PDGF-β tumors in the flank were treated as indicated. Mean tumor volume±SD are shown in 5-6 mice per group.

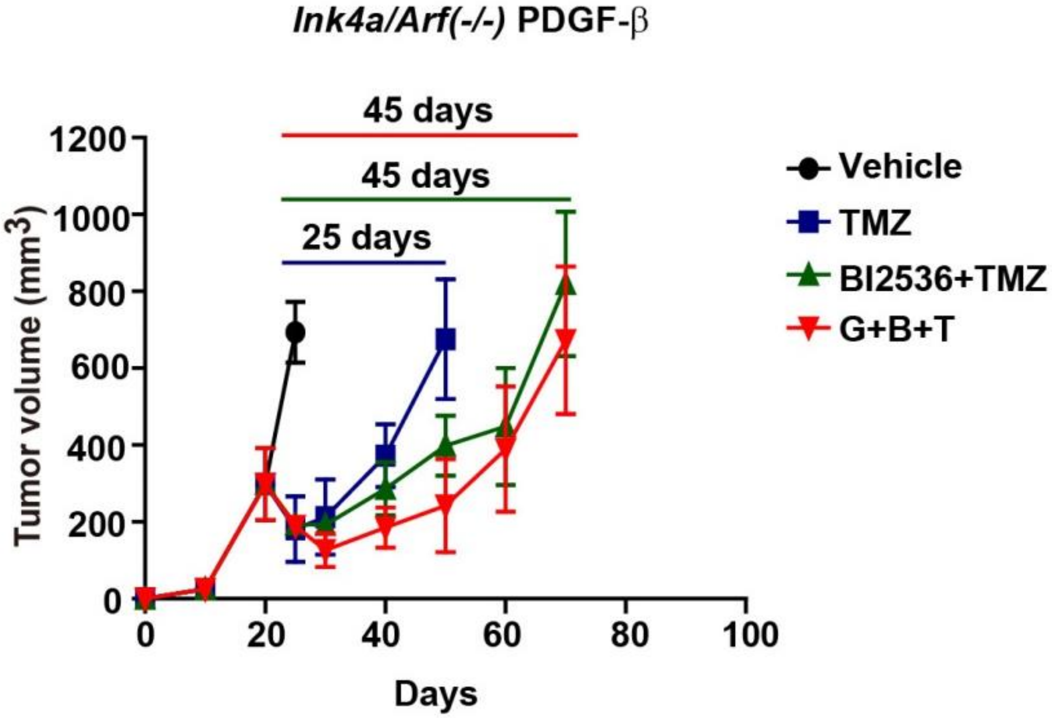

Supplemental Figure 5: Tumor growth of the subcutaneous *Ink4a/Arf(-/-)* PDGF-β allografts. Nude mice bearing established *Ink4a/Arf(-/-)* PDGF-β tumors in the flank were treated as indicated in *Methods*. T, TMZ; B, BI2536; G, Gefitinib. Mean tumor volume±SD are shown in 5-6 mice per group.
